# Fabrication and Application of Carboxymethyl Cellulose-Carbon Nanotube Aerogels

**DOI:** 10.3390/ma12111867

**Published:** 2019-06-09

**Authors:** Linyu Long, Fenfen Li, Mengying Shu, Caili Zhang, Yunxuan Weng

**Affiliations:** 1 School of Materials and Mechanical Engineering, Beijing Technology & Business University, Beijing 100048, China; 15652591908@163.com (L.L.); 13264056919@163.com (F.L.); shumengying0409@163.com (M.S.); 2 Beijing Key Laboratory of Quality Evaluation Technology for Hygiene and Safety of Plastics, Beijing Technology and Business University, Beijing 100048, China

**Keywords:** aerogel, carboxymethyl cellulose, freeze drying, compressive modulus

## Abstract

In this study, composite aerogels with excellent mechanical properties were prepared by using carboxymethyl cellulose (CMC) as raw materials, with carboxylic carbon nanotubes (CNTs) as reinforcement. By controlling the mass fraction of CNTs, composite aerogels with different CNTs were prepared, and the surface morphology, specific surface area, compressive modulus, density and adsorption capacities towards different oils were studied. Compared to the pure CMC aerogel, the specific surface areas of CMC/CNTs were decreased because of the agglomeration of CNTs. However, the densities of composite aerogels were lower than pure CMC aerogel. This is because the CNTs were first dispersed in water and then added to CMC solution. The results indicated that it was easy for the low CMC initial concentration to be converted to low density aerogel. The compressive modulus was increased from 0.3 MPa of pure CMC aerogel to 0.5 MPa of 5 wt % CMC/CNTs aerogel. Meanwhile, the prepared aerogels showed promising properties as the adsorption materials. Because of the high viscosity, liquid possesses strong adhesion to the pore wall, the adsorption capacity of the CMC aerogel to the liquid increases as the viscosity of the liquid increases.

## 1. Introduction

Carboxymethyl cellulose (CMC) is obtained by carboxylation modification of cellulose. CMC is non-toxic, biodegradable, biocompatible and soluble in water. It is the most common cellulose derivative and widely used in oil and gas exploration, food industry, textile printing, tissue engineering and other fields [1,2,3,4,5,6]. Due to the high crystallinity, amphiphilic molecular chains, strong intramolecular and intermolecular hydrogen bonds, cellulose has poor solubility in common organic solvents [7,8]. This has resulted in the preparation of regenerated cellulose aerogels becoming complex and time-consuming [9]. Because of the water solubility of CMC, the solvent replacement process can be omitted when preparing CMC aerogel [10]. The fabrication efficiency of CMC aerogel is greatly improved compared to regenerated cellulose aerogel.

Due to the inhomogeneous distribution of the CMC’s substituents, the influence of the Degree of Substitution (DS) on the mechanical strength and pore structure of the cellulose aerogel is still unclear. Surapolchai and Schiraldi prepared CMC aerogels with different DS value (0.22–0.36) and studied their compressive modulus and surface structures. They believed that the DS of CMC had a certain influence on the mechanical properties of aerogel, but the specific influence of DS on aerogel performance still needs more experimental data support [11].

The sol–gel reaction is a process in which the material transforms from the liquid sol phase to the solid gel phase. The sol–gel reaction is the most critical step in the formation of a 3D porous network structure [12,13]. Physical gels are cross-linked by physical interactions such as van der Waals forces, hydrogen bonds, hydrophobic or electronic associations, and chain entanglements [9]. However, due to the reduced number of hydroxyl groups in the molecular chain of CMC, the gelation of the cellulose solution becomes difficult and the mechanical strength of the aerogel is significantly decreased. Therefore, during the preparation of cellulose hydrogel, it is generally necessary to add inorganic reinforcement or chemical crosslinking agent to improve the mechanical properties of aerogel. Fan et al. covalently grafted lanthanide ions into the cellulose backbone by coordination with the carboxyl groups of CMC. These functionalized cellulose aerogels showed high selectivity and sensitivity with ferric ions. [14]. 

Carbon nanomaterials are often used to strengthen and functionalize cellulose aerogel materials because of their unique structure and excellent technical properties [15,16]. Huang et al. prepared a chitosan-crosslinked graphene oxide/CMC composite aerogel by mixing CMC aqueous solution with the graphene oxide suspension by a one-step process. The composite aerogel exhibited a multi-scale porous structure, and its adsorption capacities of methylene blue and chromium ions (Cr^6+^) were 3190 mg/g and 127.4 mg/g, respectively [17]. Ge et al. used the graphene oxide nanosheets to prepare the CMC composite aerogel with excellent mechanical properties. When the graphene content was 5 wt %, the compressive strength and the Young’s modulus of the composite aerogel is 349 kPa and 1029 kPa, respectively [18]. Wang et al. fabricated a graphene/CMC aerogel with stable high elasticity (4000 compression cycles at 50% strain) and excellent conductivity (86.73 S/m at 70% compression strain) using simple solution mixing and freeze-drying methods [19]. Haijian et al. prepared a superelastic CMC/carbon nanotube composite aerogel by adding unmodified single-walled and multi-walled carbon nanotubes in the CMC aqueous solution and directional freeze-drying method [20]. Carboxylic acid carbon nanotubes (CNTs) can be obtained by oxidizing carbon nanotubes [21]. The CNTs possesses large specific surface area, hydrophilicity, and good water dispersibility [22]. Therefore, CNTs are one of the most ideal inorganic additives for enhancing cellulose aerogels.

In this study, in order to further elucidate the relationship between the DS and the mechanical strength and pore structure of the cellulose aerogel, we prepared a series of CMC aerogels with different DS value (0.7, 0.9 and 1.2) by a simple freeze-drying method. Furthermore, the CMC/CNTs composite aerogels were also prepared to further improve the mechanical properties of pure CMC aerogel. The effects of DS and CNTs contents on the compressive modulus, surface structure, specific surface area and adsorption capacity of CMC aerogels were fully investigated.

## 2. Materials and Methods

### 2.1. Chemicals and Reagents

CMC (Weight-average Molecular Weight = 250000, DS = 0.7, 0.9, 1.2) and CNTs (Inner Diameter: 0.8–1.6 nm, Outside Diameter: 1–2 nm, Length: 5–30 μm, *c*_(-COOH)_ = 9.0 wt %) were purchased from Macklin Chemical Co. Ltd. (Shanghai, China). All reagents and solvents were of laboratory grade and were used without further purification.

### 2.2. Preparation of CMC Aerogel

CMC aerogel was prepared by a two-step method. First, 2 g of CMC (DS = 0.7, 0.9, or 1.2) was gradually added to 100 mL deionized water at 50 °C with vigorously stirring until a uniform transparent CMC aqueous solution obtained. Afterward, transferred the solution to a mold (diameter 15.5 mm, height 17.5 mm), allowed to stand for 24 h to form CMC hydrogel. Then, put the CMC hydrogel into a freezing dryer at −55 °C for 2 h, and vacuumed (less than 1 Pa) at this temperature for 72 h using a to obtain a CMC aerogel. To avoid water adsorption, the prepared aerogels were stored at 60 °C under vacuum. 

### 2.3. Preparation of CMC/CNTs Aerogel

**Figure 1:**
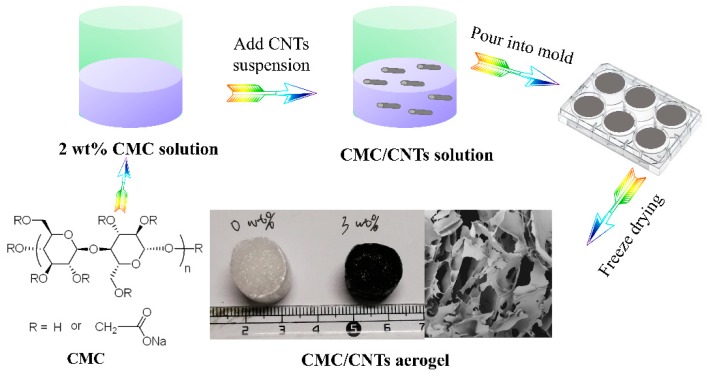
Preparation of CMC/CNTs aerogel.

Similarly, the CMC/CNTs aerogel were prepared following the same procedures of CMC aerogel, as shown in Figure 1. The only difference was after the transparent CMC solution was prepared, the pre-formed CNTs suspensions with mass fractions of 0, 1, 3, and 5 wt % (relative to the mass of cellulose) were gradually added into the solution, stirred vigorously for 2 h, poured into a mold and allowed to stand for 24 h to form CMC/CNTs hydrogel. Then CMC/CNTs aerogel was prepared by freeze-drying method.

### 2.4. Characterization

The mass (m), diameter (D), and thickness (H) of the aerogel were measured using an electronic balance (ISO9001, Beijing Sartorius Instrument Co., Ltd. Beijing, China) and a digital Vernier caliper (DL91150, Ningbo Deli Tools Co., Ltd. Ningbo, China), respectively. The volume (V) and density (ρ) of the aerogel were calculated using the mathematical calculation formulas V = π∙(D/2)^2^∙H and ρ = m/V, respectively.

The aerogel samples were first brittle cut in liquid nitrogen. After the surface was sprayed with gold, the surface morphology of the aerogel cross section was observed by scanning electron microscope (SEM) (Phenom XL, Fu Na Scientific Instrument Co., Ltd. Holland, Netherlands) at an accelerating voltage of 5 kV. The aerogel was cut into a cylindrical shape of 10 mm × 10 mm, and the compressive modulus of the aerogel was measured by a dynamic thermomechanical analyzer (DMA) (DMA7100, Hitachi Instruments Co., Ltd., Shanghai, China) at room temperature with a minimum compressive force of 10 mN and a maximum compressive force of 200 mN. Each group was repeatedly measured three times, and the results were averaged. Nitrogen adsorption experiment of aerogels were conducted using an automatic specific surface area and microporous physical adsorption instrument (MicrotracBEL Corp, BELSORP-max, Osaka-shi, Japan). First, the aerogel was pretreated at 150 °C and <10 Pa for 8 h to remove moisture and air adsorbed. Then, a nitrogen adsorption experiment was carried out in liquid nitrogen, and the specific surface area was calculated by software using a BET (Brunauer-Emmett-Teller) equation based on the aerogel adsorption-desorption curve. Three commonly used organic liquids, glycerol, polyethylene glycol-200 (PEG-200) and dimethylacetamide (DMAc) were selected for the adsorption experiments. The dimensions of the dry aerogels were 10 mm (diameter) by 8 mm (height). Aerogels were weighed and then immersed in liquid (100 mL) for 10 min. Then, the aerogel was removed from the liquid, allowed to drip dry above the liquid for 30 s to remove residual liquid on the surface, and weighed again. The adsorption capacity of the aerogel was calculated using the following formula:

C_a_ = (m_t_ − m_0_)/m_0_ (1)

where C_a_ (g/g) represents the adsorption capacity of the aerogel at 10 min and m_t_ (g) and m_0_ (g) represent the masses of the aerogel after or before adsorption, respectively.

## 3. Results and Discussion

### 3.1. The Influence of the Degree of Substitution (DS) Value on the Properties of CMC Aerogel

**Figure 2:**
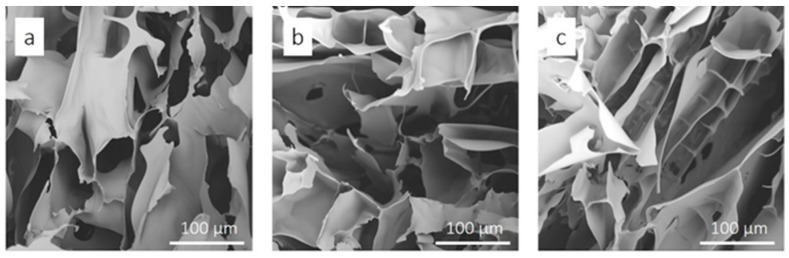
The SEM images of CMC aerogels with different DS value: (**a**) 0.7, (**b**) 0.9 and (**c**) 1.2.

The DS value of a CMC sample reflects the average number of carboxylic acid groups per glucose unit. The influence of DS of the CMC on the aerogel structures and properties was studied. SEM is one of the most common methods to analyze the surface morphology and microporous structure of porous materials. Figure 2 shows the SEM images of CMC aerogel with different DS value (0.7, 0.9 and 1.2). It is well known that the surface morphology of the aerogel largely depends on the hydrogel drying process [9,23,24]. As shown in Figure 2, the CMC aerogels prepared by freeze drying have a network structure with large pore size and sheet interconnect. This is mainly because of the formation and growth of ice crystals during hydrogel freezing process [9]. The specific surface area is the most important performance of aerogel. The static nitrogen adsorption experiment is used to analyze the BET surface area of CMC aerogels. As summarized in Table 1, the specific surface areas of the CMC aerogel are 109.94, 106.78 and 111.26 m^2^/g for DS value of 0.7, 0.9 and 1.2, respectively. The specific surface area of aerogel mainly depends on the gel drying method (freeze drying, supercritical drying or normal temperature and atmospheric drying) and parameters (for example, freezing rate and vacuum degree), and also related to the initial cellulose concentration and modification methods [23,25]. In this work, the results show that the DS value of CMC has little effect on the surface morphology and microporous structure of the aerogel.

**Figure 3:**
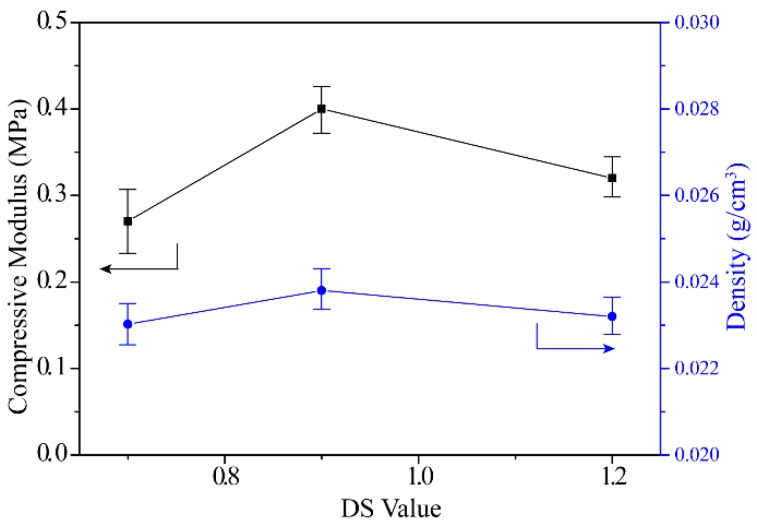
Compressive modulus and density of CMC cellulose aerogels with DS = 0.7, 0.9 and 1.2.

The compressive modulus and densities of CMC aerogels with different DS values were shown in Figure 3. For three different aerogels, the compressive modulus ranged from 0.2 to 0.4 MPa, and the densities ranged from 0.023 to 0.024 g/cm^3^. Theoretically, the reduction in the number of hydroxyl groups in the molecular chain of CMC can weaken the intermolecular forces of the cellulose aerogel, resulting in a decrease in the mechanical strength of the aerogel. However, due to the uneven distribution of substituents on the molecular chain, it is difficult to give a direct relationship between the degree of substitution and the aerogel properties. Compared with the regenerated cellulose aerogel prepared by our previous report, the density of CMC aerogel is significantly reduced. Meanwhile, the compressive modulus is also reduced [26].

The mechanical properties of cellulose aerogels are among the important properties of aerogels, which depend on the properties and concentration of the initial cellulose, crosslinks, drying methods, aerogel density, etc. [27,28]. To further enhance the mechanical properties of the CMC aerogel, different amounts of CNTs were added into the aerogel matrix with DS of 1.2. 

### 3.2. The Influence of the CNTs on the Properties of CMC Aerogel

**Figure 4:**
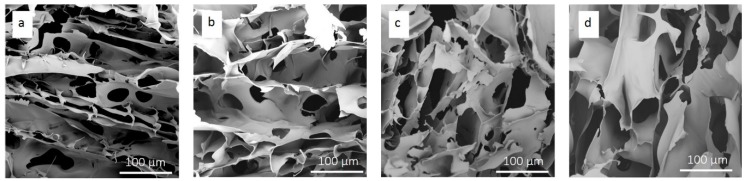
SEM images of CMC aerogel with different amounts of CNTs: (**a**) 0 wt %, (**b**) 1 wt %, (**c**) 3 wt %, (**d**) 5 wt %.

As a result of the excellent mechanical properties and water dispensability, CNTs are ideal aerogel reinforcing material. In this paper, the 2 wt % CMC solution (DS = 1.2) was mixed with 0, 1, 3 and 5 wt % of CNTs suspensions to prepare CMC/CNTs aerogel. Figure 4 shows the SEM images of CMC/CNTs composite aerogels with different amount of CNTs. CMC can be used as a binder between the CNTs to be adsorbed on CNTs to stabilize the CNTs suspension. Different amounts of CNTs suspensions were added into the CMC (DS = 1.2) solution, and CMC/CNTs aerogel was prepared by freeze drying. Table 2 summarizes the pore size distribution of aerogels which was calculated by statistical method and BET surface areas of different CNTs/CMC aerogels. 

Compared with pure CMC aerogel, the addition of CNTs suspension can reduce the CMC solution concentration. Therefore, the ability to resist the damage of ice crystal growth is weakened due to the decrease of the strength of the skeleton, and the specific surface area of the aerogel is low. However, as the amount of CNTs increases, the specific surface area of the aerogel decreases, which may be because of the agglomeration of CNTs. As a result, the addition of CNTs into the CMC matrix can reduce the pore size and surface area of the aerogel.

**Figure 5:**
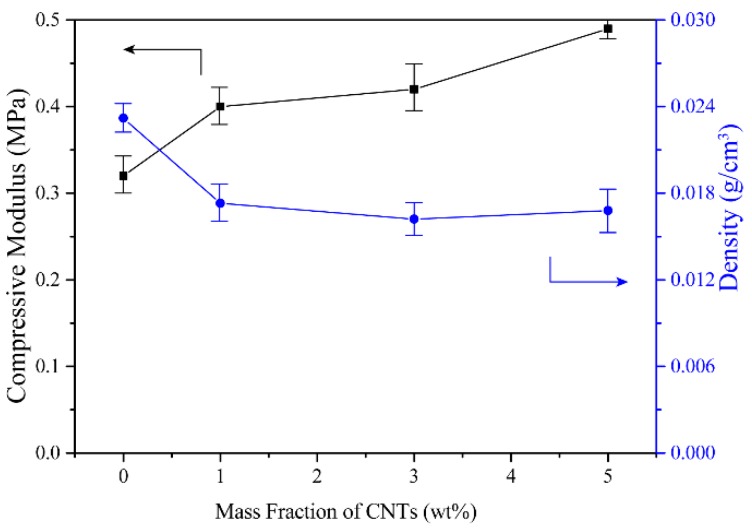
Compressive modulus and density of CMC/CNTs aerogels.

The density and compressive modulus of CMC/CNTs aerogel were shown in Figure 5. The density of pure CMC aerogel is 0.023 g/cm^3^, and the aerogel’s densities were decreased after mixing the CNTs suspension. This is mainly because the CNTs suspension diluted the CMC solution. Therefore, the aerogel density was decreased with the decreasing of the initial solution concentration. The compressive modulus of aerogel was increased from 0.3 to 0.5 MPa. The addition of CNTs improves the compression modulus of CMC aerogel and enhances its practical application performance.

### 3.3. The Adsorption Performance of Cellulose Aerogels

**Figure 6:**
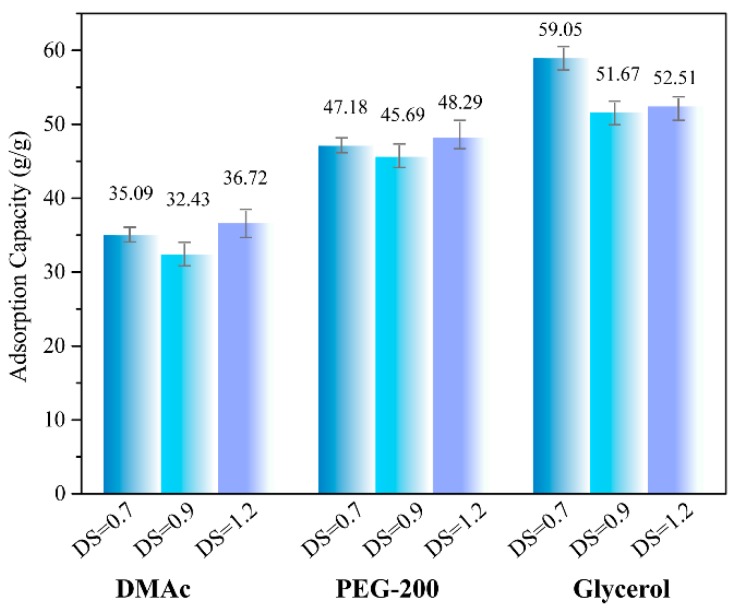
Adsorption capacity of CMC aerogels with DS = 0.7, 0.9 and 1.2.

**Figure 7:**
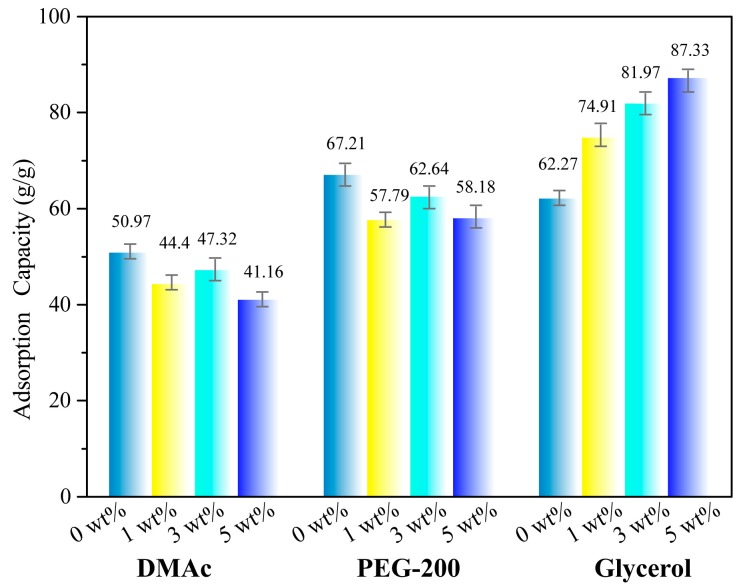
Adsorption capacity of CMC/CNTs aerogels.

Conventional adsorption materials, such as polypropylene fibers and activated carbon, are non-biodegradable and difficult to recycle after use [29,30]. Cellulose aerogels have porous network structures and abundant raw materials reserves, which have great application potential in the field of adsorbent materials [31]. Three liquids with different viscosity at room temperature, DMAc (0.92 mPa∙s), PEG-200 (22.3 mPa∙s) and Glycerol (56 mPa∙s) were selected to investigate the adsorption capacities of CMC aerogel and CMC/CNTs aerogels. Figure 6 shows that the adsorption capacities of CMC aerogel to DMAc, PEG-200 and glycerol are 32.43~36.72 g/g, 45.69~48.29 g/g and 51.67~59.05 g/g, respectively. The adsorption capacities of the aerogel increases with the increasing of the liquid viscosity. The silanized cellulose aerogel prepared by Nguyen et al. has an adsorption capacities of 4~12 g/g for toluene and vacuum pump oil [32]. The maximum adsorption capacities of microcrystalline cellulose aerogel prepared by Luo et al. to pump oil and linseed oil was 10.63 and 11.44 g/g [33]. The adsorption capacities of CMC/graphene oxide aerogel prepared by Xiang et al. to organic solvents such as methanol is 10~21 g/g [34]. As shown in Figure 7, the liquid adsorption capacities of the CMC/CNTs aerogels have a similar tendency. The effects of liquid viscosity on the adsorption properties of cellulose aerogel have two aspects. First, the liquid with lower viscosity can easily penetrate into the porous network of aerogel, and the liquid with high viscosity has a large adhesion to the pore wall of aerogel and a high liquid retention rate.

## 4. Conclusions

In this work, CMC aerogels with different porous structure were prepared by simple cellulose dissolution, solution mixing, and freeze-drying methods. The effects of the DS value and CNTs amount on the surface structure, compressive modulus, density, specific surface area and adsorption capacities for CMC aerogels were studied. The results show that:

(1) The DS value has little effect on the surface structure, mechanical properties and density of the CMC aerogels due to the uneven distribution of the substituents.
(2) Because of the agglomeration of CNTs, the specific surface area of the aerogels decreases with the increasing of CNTs amount. The aerogel densities were decreased after adding the CNTs suspension into the CMC matrix because of decreasing the solution concentration. The compressive modulus of aerogel was increased from 0.3 MPa of pure CMC aerogel to 0.5 MPa of 5 wt % CNTs/CMC aerogel.
(3) Because the high viscosity liquid possesses strong adhesion to the pore wall, the adsorption capacity of the CMC aerogel to the liquid increases as the viscosity of the liquid increases.

## Figures and Tables

**Table 1:** The BET surface area for different DS value CMC aerogels.

DS Value	0.7	0.9	1.2
BET surface area (m^2^/g)	109.94	106.78	111.26

**Table 2:** The pore sizes distribution range and BET surface areas for different CNTs amounts of CMC (DS = 1.2) aerogels.

CNTs Amounts (wt %)	0	1	3	5
Pore size distribution (μm)	30.6~152.5	9.71~128	8.7~118	16.6~132.4
BET surface area (m^2^/g)	111.26	100.56	70.21	66.81
